# Development of lab score system for predicting COVID-19 patient severity: A retrospective analysis

**DOI:** 10.1371/journal.pone.0273006

**Published:** 2022-09-09

**Authors:** Arnab Sarkar, Surojit Sanyal, Agniva Majumdar, Devendra Nath Tewari, Uttaran Bhattacharjee, Juhi Pal, Alok Kumar Chakrabarti, Shanta Dutta

**Affiliations:** ICMR- National Institute of Cholera and Enteric Diseases, Beliaghata, Kolkata, India; Regional Medical Research Centre Bhubaneswar, INDIA

## Abstract

**Aim:**

To develop an accurate lab score based on in-hospital patients’ potent clinical and biological parameters for predicting COVID-19 patient severity during hospital admission.

**Methods:**

To conduct this retrospective analysis, a derivation cohort was constructed by including all the available biological and clinical parameters of 355 COVID positive patients (recovered = 285, deceased = 70), collected in November 2020-September 2021. For identifying potent biomarkers and clinical parameters to determine hospital admitted patient severity or mortality, the receiver operating characteristics (ROC) curve and Fischer’s test analysis was performed. Relative risk regression was estimated to develop laboratory scores for each clinical and routine biological parameter. Lab score was further validated by ROC curve analysis of the validation cohort which was built with 50 COVID positive hospital patients, admitted during October 2021-January 2022.

**Results:**

Sensitivity vs. 1-specificity ROC curve (>0.7 Area Under the Curve, 95% CI) and univariate analysis (p<0.0001) of the derivation cohort identified five routine biomarkers (neutrophil, lymphocytes, neutrophil: lymphocytes, WBC count, ferritin) and three clinical parameters (patient age, pre-existing comorbidities, admitted with pneumonia) for the novel lab score development. Depending on the relative risk (p values and 95% CI) these clinical parameters were scored and attributed to both the derivation cohort (n = 355) and the validation cohort (n = 50). ROC curve analysis estimated the Area Under the Curve (AUC) of the derivation and validation cohort which was 0.914 (0.883–0.945, 95% CI) and 0.873 (0.778–0.969, 95% CI) respectively.

**Conclusion:**

The development of proper lab scores, based on patients’ clinical parameters and routine biomarkers, would help physicians to predict patient risk at the time of their hospital admission and may improve hospital-admitted COVID-19 patients’ survivability.

## Introduction

The emergence of Coronavirus 2019 disease (COVID-19), an acute respiratory infectious disease has frightened the world with its infectivity and ability for rapid evolution. Since its outbreak in Wuhan, China in 2019 [1, 2], it had been expanded across the world and caused three or four COVID-19 infection waves in the last two years. Sequence analysis confirmed its closeness to bat-derived Yunan/ RaTG13/ 2013 coronavirus strain [3], but its route of evolution or emergence has not been elucidated yet. With the rapid emergence or wave of COVID-19 infection, it became a global challenge for hospitals to provide adequate medical resources to both COVID-19 positive and non-COVID-19 patients [4, 5]. During the COVID-19 pandemic, all the hospitals were overwhelmed with positive patients [6] and in May 2021, India registered 4,00,000 daily infection cases and 5000 daily mortality cases due to COVID-19 infection [7]. Among all the Indian states, Maharashtra, Kerala, Karnataka, Andhra Pradesh, Tamil Nadu, Delhi, Uttar Pradesh, and West Bengal [8] were affected most with the emergence of delta variant B.1.617.2 [9]. In our previous study, we compared the second wave with the first wave of the COVID-19 in India and concluded that the rapid rise of the delta variant was one of the reasons for rapid infection [10]. Furthermore, the delta variant reported more patient hospitalization and Intensive Care Unit (ICU) admission than the alpha variant of concern [11]. In the third wave of COVID-19 infection in India, the union health secretary announced that the hospitalization cases in the third wave were 5–10% as compared to 20–23% in that of the second wave [12] and the requirement of oxygen was reduced to 17.6% in the omicron wave as compared to 74% in the delta wave. Surprisingly, the ’Our world data’ repository showed that the number of US hospital and ICU admission recorded higher in the omicron wave than that of the delta wave [13]. As the infectivity of omicron variants was 3–6 times higher than the delta variant [14], it was also predicted that the number of hospitalization might be increased due to its longer prevalence.

For early prediction of the risk of COVID-19 positive patient severity or mortality, several studies had been performed in the recent past and among them, a few retrospective studies developed a novel scoring system based on patients’ clinical and biological parameters [15–17]. However, with the rapid emergence of SARS-CoV-2 variants, the patient’s clinical manifestation was also changed continuously. Wrenn et al. reported that delta variants caused more number of patients with dyspnea than omicron variants [18]. In our previous study, we presented that the median age of COVID-19 positive patients in India was also shifted from younger (11–45 years) to older patients (31–60 years) in the 2nd wave as compared with the 1st wave of COVID-19 infection [10]. Thereby, a universal scoring system needs to be developed for predicting the risk of COVID-19 positive patient severity irrespective of any SARS-CoV-2 variant infection. For developing proper lab scores, we have included hospital admitted COVID-19 positive patient data in the period of November 2020-September, 2021 as derivation cohort and validated the developed score with the COVID-19 patient data admitted in October 2021-January 2022 to hospitals.

To manage the catastrophe in hospital admission amid the COVID-19 pandemic, a novel lab score generation may be useful for predicting patient severity during the time of hospital admission. In this study, a novel scoring system is developed based on clinical and routine biological parameters of Indian COVID-19 patients, collected from three West Bengal hospitals viz. B.R. Singh Hospital, Sealdah, Kolkata; Calcutta Medical Research Institute (CMRI), Kolkata; and AMRI Hospital, Salt-lake, Kolkata. As our lab score is based on patients’ initial biological and clinical parameters, measuring the lab score for each patient at the time of hospital admission would pre-determine severe patients from non-severe patients and would provide intensive care facilities to improve COVID-19 patients’ survivability.

## Materials and methods

### Study design and data sources

With the emergence, rapid spread and related case fatality a network of COVID-19 bio-repositories were established in India by Indian Council of Medical Research (ICMR) to create a COVID-19 resource that can be used by scientists, researchers and industry in the current pandemic scenario or in future. Under this project, the bio-repository established at ICMR-NICED collected samples and clinical data from the COVID-19 positive patients admitted in three hospitals of West Bengal viz. B.R. Singh Hospital, Sealdah, Kolkata (B.R. Singh Hospital), The Calcutta Medical Research Institute (CMRI) and AMRI Hospital, Salt Lake, Kolkata.

For the current study, anonymized clinical data were collected and analyzed retrospectively from the records of the COVID-19 biorepository at ICMR-NICED. All hospital admitted COVID-19 positive patients’ initial laboratory biomarkers (hematological, inflammation, biochemical, coagulation) and clinical features were included in our retrospective study.

In order to pre-determine the potent biomarkers for the COVID-19 patient severity, all hematological (hemoglobin, neutrophil, lymphocytes, PCV, platelets, WBC count), inflammatory (Ferritin, pro-calcitonin, CRP, ESR), biochemical (creatinine) and coagulation biomarkers (D-Dimer, LDH) at the time of hospital admissions were categorized into the recovered and deceased patient group. Furthermore, for determining patient severity, patient’s age, gender, comorbidities and pneumonia status were also incorporated in the current study. This study was performed with approval from the Institutional Ethics Committee of ICMR- National Institute of Cholera and Enteric Diseases, Kolkata (A-1/2020-IEC dated July 27, 2020).

### Derivation cohort and validation cohort

The derivation cohort was constructed with hospitalized patients data (total n = 355, recovered n = 285, dead n = 70), collected from three West Bengal hospitals during November 2020-September, 2021 (S1 Data). All initial biological parameters and clinical characteristics of the derivation patient cohort were included for the lab score construction. For validating the assigned scores, the validation cohort was built with the clinical details of 50 COVID-19 positive patients (recovered n = 20, dead n = 30), admitted in BR Singh Hospital during the period October 2021-January 2022 (S2 Data).

### Score construction

For the development of the predicting score of patient severity, a univariate analysis of all potent biological parameters was conducted. Among them, statistically significant parameters were included in the relative risk regression analysis for the score assignment. Depending on the relative risk (95% CI) of patient’s clinical characters and biological parameters with patient’s severity or mortality, score ‘0’, ‘1’, ‘2’, ‘3’, ‘4’ was assigned. The efficiency of the predicted score of the derivation cohort was validated further with the derivation cohort by ROC curve analysis.

### Statistical analysis

For evaluating the potent biomarkers responsible for predicting in-hospital mortality, the receiver operating characteristic (ROC) curve was used. AUC of each biomarker above 0.7 was considered as effective for pre-diagnosing patient severity. The cut-off point for each potent biomarker was also calculated from sensitivity vs. 1-specificity plot. The level of significance of all categorical variables like patient age, number of patients with comorbidities or pneumonia (mild, severe) and numerical variables between recovered and deceased groups were calculated by Fischer’s test and student’s t-test respectively. For evaluating the association of biological parameters with the patient’s in-hospital mortality, relative risk or risk ratio was calculated. A risk ratio > 1 indicated that patients’ numerical or categorical markers during admission were likely to be associated with in-hospital mortality. Confidence interval (CI) was set at 95% and online risk ratio calculator, https://www.medcalc.org/calc/relative_risk.php was used for the relative risk analysis.

ROC curve analysis was again performed for testing the accuracy of predicting severity between cured and deceased patients. ROC plot and AUC curve of each biomarkers, derivation and validation cohort were plotted by the software Graphpad Prism 5.0.

## Results

**Fig 1:**
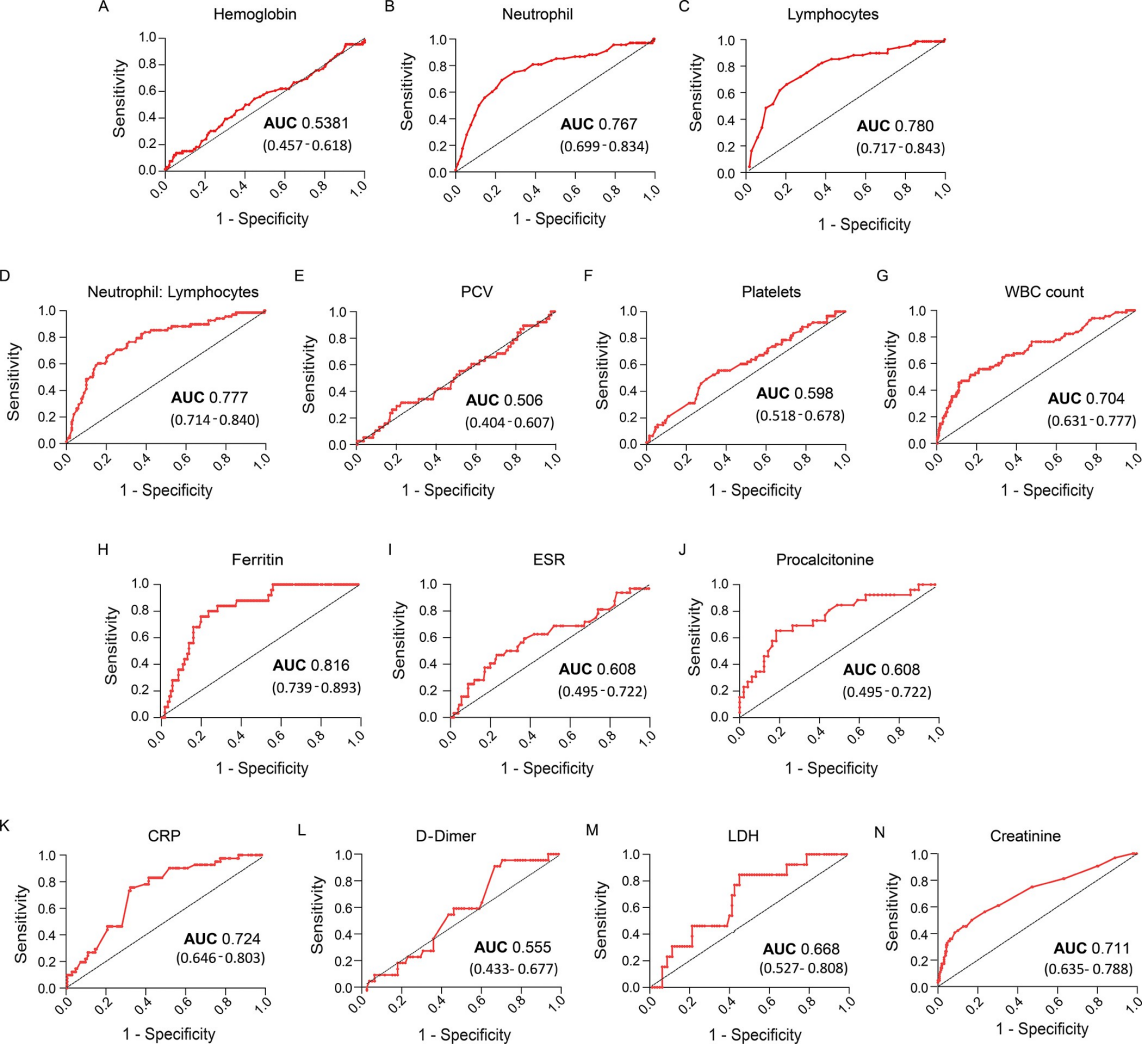
A-F) Reactive OC curve of all the routine biomarkers (hemoglobin, neutrophil, lymphocytes, neutrophil: lymphocytes, PCV, platelets, WBC count, Ferritin, ESR, procalcitonin, C-reactive protein, d-dimer, LDH, creatinine) were plotted based on sensitivity vs. 1-specificity. AUC (95% CI) value depicted the ability of each biomarker to discriminate between in-hospital mortality and recovery.

**Fig 2:**
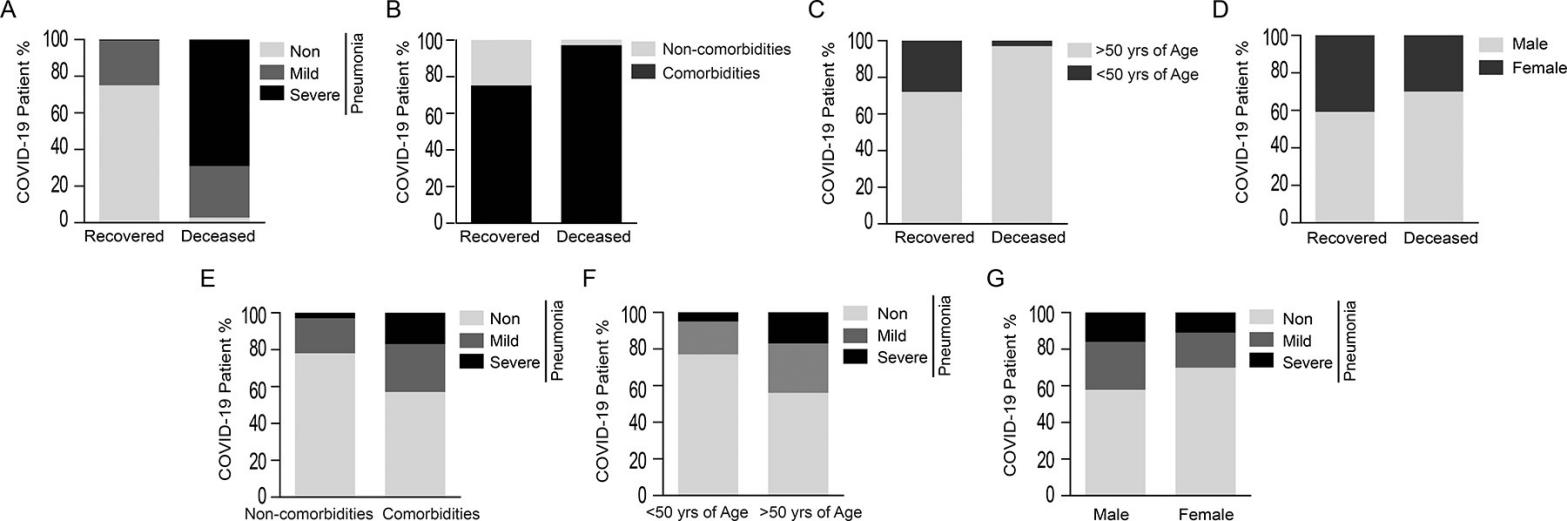
A-D) Stacked bar chart represented the effects of pneumonia (no, mild, severe), pre-existing comorbidities, patient age (below and above 50 years) and gender (male and female) at the time of hospital admission on the COVID-19 patient’s recovery and mortality. E-G) stacked bar chart displaying the percentage of COVID positive patients admitted with pneumonia (no, mild, severe) with the pre-existing comorbidities, patients’ age and gender.

For this analytical study, a derivation cohort (n = 355) was constructed by including information from COVID-19 positive patients at the time of their admissions in three hospitals of West Bengal. All the routine biomarkers tested during hospital admission were categorized into four groups-hematological (hemoglobin, lymphocytes, neutrophil, platelets, PCV), inflammation (ferritin, procalcitonin, ESR, CRP), biochemical (creatinine) and coagulation biomarkers (D-Dimer, LDH) (S1 Data). To test whether any biomarker has the potential in determining in-hospital mortality, sensitivity vs. 1-specificity of recovered and deceased patients were plotted. The analysis showed that among all markers neutrophils, lymphocytes, neutrophils: lymphocytes, WBC count, ferritin, CRP and creatinine were scored above 0.7 AUC (p<0.0001) (Fig 1A–1F). In this study, we included these clinical parameters for predicting patient severity or mortality. Statistical analysis revealed that severe pneumonia (p<0.0001) (Fig 2A), pre-existing comorbidity (p<0.0001) (Fig 2B) and >50 yrs. patient’s age (Fig 2C) were three potent factors (p<0.0001) in deceased COVID-19 patients as compared with recovered patients. However, patient gender had no such impact on in-hospital mortality (Fig 2D) or patient pneumonia (Fig 2G). Fischer’s test analysis showed that patient comorbidities or age above 50 years had a significant impact on patient pneumonia (Fig 2E, 2F), thereby linking severe pneumonia with pre-existing comorbidity and patients age >50 years. In S1 Table, a univariate analysis (p<0.0001) revealed that five routine biomarkers and three clinical parameters exhibited significant differences (p-value <0.01) between recovered and deceased patients. However, due to the inadequate availability of procalcitonin reports in the derivation cohort, it was excluded from this study. By using risk ratio analysis of significant parameters in the derivation cohort, a score was introduced. Here, the above or below cut-off point (by using the Youden index) of each biomarker was used in the risk ratio evaluation. Patient with pre-existing comorbidity or >635.7ng/ml ferritin was assigned as score ‘1’. Depending on the risk ratio, patient age groups of ≤ 57yrs, >57yrs- <69 yrs and ≥69 yrs were scored as ‘0’ (p < 0.0001), ‘1’ (p = 0.6353), ‘2’ (p < 0.0001) respectively. Patient with mild and severe COVID-pneumonia was endorsed with ‘1’(p = 0.5490) and ‘4’ (p < 0.0001) respectively. Above the cut-off of other regular biomarkers like WBC count, neutrophil, lymphocytes, neutrophil: lymphocytes (N: L) were assigned with a score ’3’ (p < 0.0001) (S2 Table).

**Fig 3:**
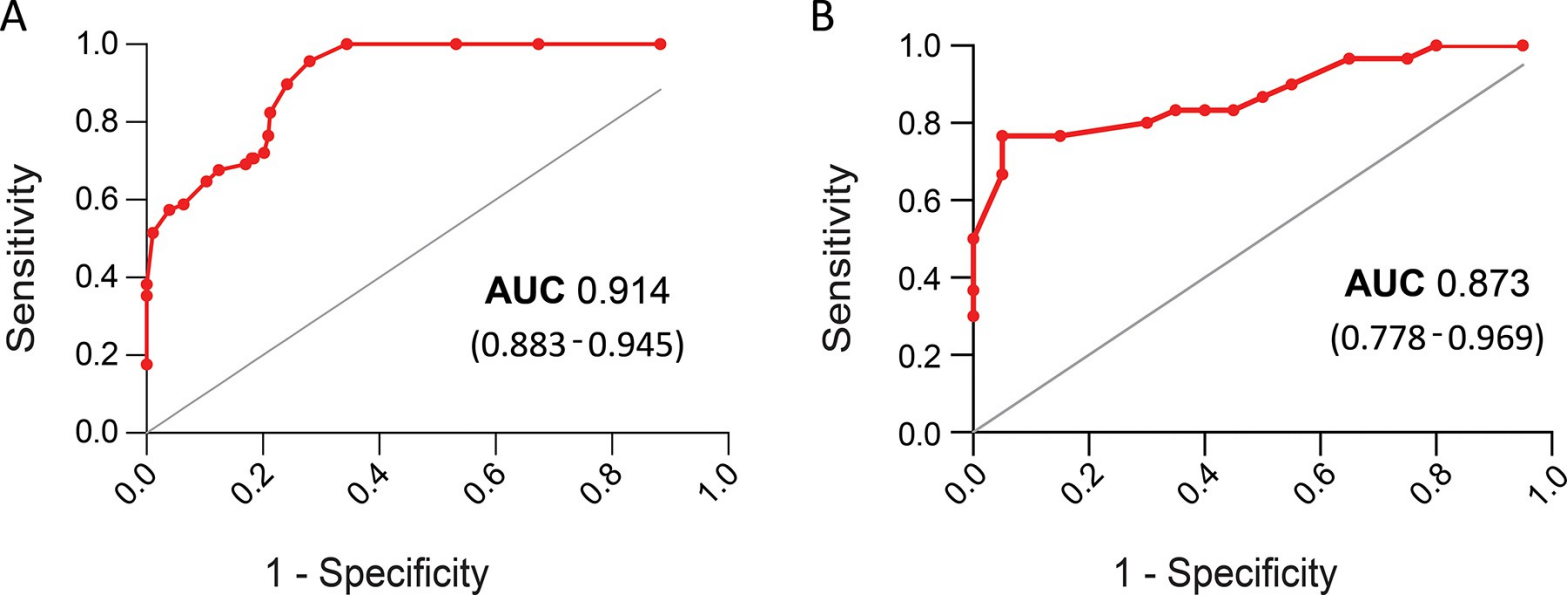
ROC curve of derivation cohort (A) and validation cohort (B) was plotted. High AUC (>0.7) showed the lab score efficiency in predicting patient severity or poorer outcomes.

After generating the scoring system, the whole derivation cohort was, scored accordingly and the ROC curve was plotted (Fig 3A). AUC 0.914 (0.883–0.962, 95% CI, p< 0.0001) confirmed that the lab score could efficiently predict in-hospital mortality in the derivation cohort. To validate whether the lab score would determine patient severity during admission and irrespective of any SARS-CoV-2 variant, a COVID-19 patient data of October 2021-January 2022 was collected from B.R. Singh hospital (S2 Data). A total of 50 patient data was used in the preparation of the validation cohort (S3 Table) and ROC curve analysis of the cohort generated AUC 0.873 (0.778–0.969, 95% CI, p< 0.0001), which was quite similar to the AUC of the derivation cohort score (Fig 3B).

## Discussion

This retrospective study determined that during COVID-19 patient admission in three hospitals of West Bengal, five potent biomarkers (neutrophil count, lymphocytes count, neutrophil: lymphocyte ratio, WBC count and ferritin) and three clinical conditions (>50 years age, patient comorbidities and admission with pneumonia) are significant cues in predicting patient severity and in-hospital mortality. Literature analysis showed that patient age [19], comorbidities [20] and admission with pneumonia [21] played substantial role in patient severity or risk of mortality. Our retrospective analysis found that severe pneumonia was one of the key factor for patient mortality as 49 out of 70 dead patients and 30 out of 30 dead patients were admitted to hospital with mild/ severe pneumonia in our derivation and validation cohort respectively. According to WHO, the clinical manifestation of mild pneumonia comprises fever, cough, dyspnea and breathing with SpO2 ≥ 90%. Whereas, severe pneumonia in adults have similar mild pneumonia symptoms with SpO2< 90% [22] and >30 breaths/ min [23]. Mitsi et al. reported that *Streptococcus pneumoniae* co-infection with SARS-CoV-2 facilitated evading host immunity and expedited inflammatory responses [24]. Additionally, the Centers for Disease Control and Prevention claimed that the pneumonia vaccine could prevent COVID-19 pathogenicity effectively [25].

In a clinical investigation, Du et al. 2020 showed that older (>65 yrs) patients were more susceptible to COVID-19 pneumonia [26]. Our study explored that (>68.7±10.6 yrs.) older COVID-19 patients were at high risk than middle-aged or younger patients. In the derivation cohort, >50 yrs older patients were more susceptibility to severe pneumonia (p<0.05) as compared to <50 yrs old group. Likewise, higher in-hospital mortality was observed in COVID-19 patients admitted with pre-existing comorbidities like hypertension, diabetes, asthma, cardiovascular diseases, etc. (p<0.05).

Despite any direct association of WBC count or lymphocytes [27], high neutrophil count or neutrophil: lymphocyte ratio were considered as a determining factor of viral infection or infection-related acute respiratory failure [28]. Moreover, high neutrophil count and lymphocytes dysfunction triggered hyper-inflammation, followed by acute respiratory disease syndrome (ARDS) [29]. Our retrospective analysis revealed that below the cut-off of lymphocyte count (<9.5%, p<0.0001) and above the cut-off of WBC (>12.55x103/μL), neutrophil count (> 85.50%, p<0.0001) and neutrophil: lymphocyte ratio (>8.47, p<0.0001) contributed in the early detection of COVID-19 patient severity during hospital admission. Along with this, ferritin was another biomarker of concern and ferritin level >635.7ng/ml might be one of the key factors for determining patient severity. Ferritin is an indirect iron store marker commonly used for iron deficiency diagnosis [30]. However, increased ferritin level or hyperferritinemia in the patient serum was associated with high cytokine, growth factor secretion, damaging hepatocyte cells in liver disease [31, 32] and pulmonary severity [33]. High ferritin level in the patient serum was also linked with patient pneumonia and could be used as a diagnostic or prognostic marker for patient severity [34]. In the derivation patient cohort, Fischer’s test showed a significant association of level of ferritin with severe patient pneumonia (p<0.01).

The development of lab scores based on patients’ clinical biomarkers is not new. For managing the risk of patient severity, several previous studies developed lab scores for infectious diseases viz. Respiratory syncytial virus (RSV) infection in infants [35] and H1N1 virus infection [36]. Since the emergence of SARS-CoV-2, several lab scores were developed based on the finding of clinical laboratory biomarkers of hospital admitted patient cohort [37–40]. Univariate analysis of both the derivation (S1 Table) and the validation cohort (S3 Table) showed that patients with older age, comorbidities, admission with pneumonia, neutrophil and lymphocyte count might be a significant factor for COVID-19 severity prediction (p<0.05). As these clinical biomarkers are common diagnostic parameters, they may have the potential to predict the severity risk for other infectious diseases. However, the cut-off may be specific for each infectious disease. As we derived the cut-off for each parameter from the retrospective analysis of COVID-19 patients only, these scores may not be functional for other infectious diseases. On 17th May 2021, the Ministry of Health and Family Welfare, Govt. of India announced clinical guidelines of hospital admission, declaring that moderate and severe COVID-19 patients may admit to the hospital ward and Intensive Care Unit (ICU) respectively [41]. However, Indian hospitals faced serious challenges in providing medical resources to admitted patients during the delta COVID-19 wave as at the peak time the infection rate crossed over 4,00,000 cases per day. With the rapid surge of delta virus infection, hospitals started running out of ICU beds and oxygen supply across the country [42, 43]. Therefore a proper lab scores based on the relative risk of patient’s clinical parameters and routine biomarkers would be an alternative approach for hospital managements to categorize moderate and severe illness at the time of admission. Moreover, a proper lab score based on clinical data from the Indian hospitals needs to be developed or revised time to time. Here, we included clinical parameters of Indian COVID-19 patients for the novel lab score development. The ROC curve analysis of both derivation (AUC 0.914) and validation (AUC 0.873) cohort confirmed the lab score efficiency for predicting patients’ poorer outcomes at the time of hospital admission.

Our study showed that our lab score worked for both the patient cohorts collected from admitted in the period of delta and omicron COVID-19 infection wave in India, and determine the patient severity efficiently at two different period of COVID-19 infection, it may be able to predict patient severity in future COVID-19 infection waves.

Besides its significance, this retrospective study has several limitations. The first limitation of this retrospective study is its small cohort size and unavailability of all the routine biomarker reports of COVID-19 positive patients at the time of admission. We collected information on all the clinical and biological parameters of COVID-19 positive patients from three west Bengal hospitals and compiled them to get a standard cohort size for the analysis. Several studies explored the relation of pre-existing diabetes [44], hypertension [45] and cardiovascular diseases [46] with patient severity or mortality. However, due to the small cohort size, we can’t analyze all the comorbidities separately and generalize the score of patients with pre-existing comorbidities as score ’1’ (RR- 1.302, P<0.0001). Secondly, as infection of SARS-CoV-2 variants were different and caused different clinical manifestations, a generalized lab score may not be enough for predicting patient severity. For example, Delta COVID-19 variant infection caused acute hypoxic respiratory failure [47] and more patients were admitted to the ICU for oxygen supplements, whereas COVID-19 patients infected with Omicron and other variants reported fewer effects on the blood oxygen level [48]. Thirdly, admitted COVID-19 patients were treated with antivirals and some of them were vaccinated or re-infected which may lower in-hospital mortality and could make an impact on the lab score analysis. Finally, the risk of multi-organ failure in post-COVID patients suggested a long follow-up period to evaluate the impact of this score.

## Conclusion

This is a useful study for predicting the mortality risk of COVID-19 positive patients during their admissions. Development of a proper lab score, based on patients’ routine biomarkers and clinical parameters of a patient, will help to categorize admitted patients by predicting their outcomes. A high score of a COVID-19 positive patient admitted with severe pneumonia, ≥69 years old, pre-existing comorbidity, high ferritin (>635.7ng/ml) and NLR (>8.47) would be associated with the worst consequence.

## Supporting information

S1 Table Univariate analysis of recovered and deceased patients with COVID-19 in retrospective data analysis (derivation cohort). (DOCX)

S2 Table Relative risk (RR) regression analysis of hospitalized patient-mortality with COVID-19 infection. (DOCX)

S3 Table Univariate analysis of recovered and deceased patients of COVID-19 in validation cohort. (DOCX)

S1 Data All initial biological parameters and clinical characteristics data of hospitalized patients (total n = 355, recovered n = 285, dead n = 70), collected from three West Bengal hospitals during November 2020-September, 2021 was included in the derivation cohort. (XLSX)

S2 Data Initial biological parameters (Age, admission with pneumonia, comorbidities) and clinical characteristics (Neutrophil. Lymphocytes, WBC count, Ferritin) data of hospitalized patients (total n = 50, recovered n = 20, dead n = 30), collected from B.R. Singh hospital during October 2021-January 2022 was included in the validation cohort. (XLSX)
