# Supplementary material for: Development of lab score system for predicting COVID-19 patient severity: A retrospective analysis
Source: PLoS One. 2022 Sep 9;17(9):e0273006. doi: 10.1371/journal.pone.0273006 (PMC9462772; doi:10.1371/journal.pone.0273006)
Supplement: S1 Table — (DOCX) [file pone.0273006.s001.docx]

**S1 Table**

Univariate analysis of recovered and deceased patients with COVID-19 in retrospective data analysis

| **Predictors and biomarkers** | **Recovered (n=285)** | **Deceased (n=70)** | **P value** |
| --- | --- | --- | --- |
| Age years (Mean ± SD) | 57.3 ± 15.4 | 68.7 ± 10.6 | <0.0001 |
| Female patients, n (%) | 119 (40.33) | 21 (28) | 0.055 |
| Admitted with pneumonia, n (%) | 73 (25) | 69 (97.1) | <0.0001 |
| Admitted with comorbidities, n (%) | 218 (74.6) | 69 (97.1) | <0.0001 |
| Hemoglobin gm/dl (Mean) | 11.78 | 11.46 | 0.252 |
| Neutrophil % (Mean) | 77.6 | 86.4 | <0.0001 |
| Lymphocytes % (Mean) | 17.83 | 9.39 | <0.0001 |
| Neutrophil : lymphocytes (Mean) | 7.21 | 15.54 | <0.0001 |
| PCV % (Mean) | 36.03 | 36.12 | 0.937 |
| Platelet count x 10^3^ /µL (Mean) | 225 | 190 | 0.021 |
| WBC count x 10^3^ /µL (Mean) | 9.8 | 14.57 | <0.0001 |
| Ferritin ng/ml (Mean) | 529.4 | 1037 | 0.003 |
| ESR mm/hour (Mean) | 52.04 | 63.97 | 0.101 |
| Procalcitonin ng/ml (Mean) | 1.4 | 10.9 | 0.0058 |
| CRP (Mean) | 21.7 | 60.9 | 0.278 |
| D-Dimer ng/dl (Mean) | 365.8 | 289.6 | 0.233 |
| LDH IU/L (Mean) | 285.9 | 328.7 | 0.399 |
