# Supplementary material for: Development of lab score system for predicting COVID-19 patient severity: A retrospective analysis
Source: PLoS One. 2022 Sep 9;17(9):e0273006. doi: 10.1371/journal.pone.0273006 (PMC9462772; doi:10.1371/journal.pone.0273006)
Supplement: S2 Table — (DOCX) [file pone.0273006.s002.docx]

**S2 Table**

Relative risk (RR) regression analysis of hospitalized patient-mortality with COVID-19 infection

| **Predicting markers** | **Relative Risk or RR (95% CI)** | **P-value** | **score** |
| --- | --- | --- | --- |
| Age  ≤ 57 years  >58 -<69 years  ≥ 69 years | 0.263 ( 0.142-0.490)  1.107 ( 0.7265-1.6878)  2.288 ( 1.734-3.019) | P < 0.0001  P = 0.6353  P < 0.0001 | 0  1  2 |
| Admitted with pneumonia-  Mild  Severe | 1.138 (0.745-1.738)  201.5211 (28.3-1434.7) | P= 0.5490  P < 0.0001 | 1  4 |
| Admitted with comorbidities | 1.302 (1.205-1.407) | P < 0.0001 | 1 |
| Neutrophil > 85.50 % (Cut-off point) | 2.902 (2.261-3.725) | P < 0.0001 | 3 |
| Lymphocytes <9.5% (Cut-off point) | 3.125 (2.338-4.176) | P < 0.0001 | 3 |
| Neutrophil : lymphocytes >8.47 (Cut-off point) | 2.806 (2.183-3.607) | P < 0.0001 | 3 |
| WBC count x 10^3^ >12.55/µL (Cut-off point) | 2.584 (1.926-3.467) | P < 0.0001 | 3 |
| Ferritin ng/ml >635.7 (Cut-off point) | 1.366 (1.215-1.535) | P < 0.0001 | 1 |
| Total score = 20 | | | |
