# Supplementary material for: Development of lab score system for predicting COVID-19 patient severity: A retrospective analysis
Source: PLoS One. 2022 Sep 9;17(9):e0273006. doi: 10.1371/journal.pone.0273006 (PMC9462772; doi:10.1371/journal.pone.0273006)
Supplement: S3 Table — (DOCX) [file pone.0273006.s003.docx]

**S3 Table**

Univariate analysis of recovered and deceased patients of validation cohort

| **Predictors and biomarkers** | **Recovered (n=20)** | **Deceased (n=30)** | **P value** |
| --- | --- | --- | --- |
| Age years (Mean ± SD) | 61.05 ± 12.66 | 70.8 ± 15.47 | 0.0401 |
| Admitted with pneumonia, n (%) | 9 (45) | 30 (100) | < 0.0001 |
| Admitted with comorbidities, n (%) | 17 (74.6) | 27 (97.1) | < 0.0001 |
| Neutrophil % (Mean) | 84.05 | 88.9 | 0.0463 |
| Lymphocytes % (Mean) | 11.05 | 6.96 | 0.0126 |
| Neutrophil : lymphocytes (Mean) | 11.22 | 17.37 | 0.084 |
| WBC count x 10^3^ /µL (Mean) | 12.28 | 14.04 | 0.237 |
| Ferritin ng/ml (Mean) | 847.7 | 903.8 | 0.1 |
